# Targeted Thrombolysis via CCR2‐Engineered Macrophage‐Mimicking Microbubbles Safely Ablates Venous, Arterial, and Microvascular Thrombosis

**DOI:** 10.1002/advs.202524002

**Published:** 2026-03-13

**Authors:** Buying Li, Changjin Lu, Shijie Gao, Dandan Chen, Xue Heng, Kibret Mequanint, Malcolm Xing, Gaoxing Luo, Haisheng Li

**Affiliations:** ^1^ Institute of Burn Research Southwest Hospital State Key Laboratory of Trauma and Chemical Poisoning Chongqing China; ^2^ College of Bioengineering Chongqing University Chongqing China; ^3^ Department of Chemical and Biochemical Engineering University of Western Ontario London ON Canada; ^4^ Department of Mechanical Engineering University of Manitoba Winnipeg MB Canada

**Keywords:** inflammatory microenvironment, macrophage membrane, microbubble, Thrombosis, ultrasound

## Abstract

The clinical management of thrombosis, a primary cause of death worldwide, is hampered by the limitations of current thrombolytic agents, including short half‐life and high risk of off‐target bleeding. Here, we report the design and validation of an intelligent, inflammation‐targeting microbubble for precise thrombolysis. We first engineered macrophages to overexpress the C─C chemokine receptor 2 (CCR2) via lentiviral transfection. Membranes derived from these cells were then used to functionalize a liposomal structure, co‐encapsulating the thrombolytic drug urokinase (UK) and a phase‐change perfluoropropane gas. These resulting biomimetic microbubbles (termed UK@CCR2/MBs) were designed to navigate the vasculature and home in on thrombotic sites by binding to the highly expressed monocyte chemoattractant protein‐1 (MCP‐1) via the CCR2 receptor. Upon arrival at the target, localized low‐frequency ultrasound was applied to trigger acoustic droplet vaporization, leading to microbubble disruption and spatiotemporally controlled UK release. Extensive in vitro and in vivo evaluations, including in animal models of deep vein, carotid artery, and microcirculatory thrombosis, confirmed that UK@CCR2/MBs achieve superior thrombolytic efficacy and specific targeting with an excellent safety profile. This macrophage‐mimicking, ultrasound‐responsive system represents a sophisticated theranostic platform for the non‐invasive and targeted treatment of thrombotic diseases.

## Graphical abstract

CCR2‐Enhanced Macrophage Membrane–Camouflaged Microbubbles for MCP‐1–Mediated Thrombus Targeting and Ultrasound‐Triggered Urokinase Release.

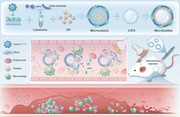

## 1 Introduction

Accounting for one in four deaths globally, thrombosis stands as a paramount challenge to public health [1, 2]. Whether forming in arteries or veins, a thrombus can cause local vessel occlusion or detach and embolize, culminating in devastating clinical events like heart attacks, strokes, and pulmonary embolisms [3]. This long‐standing threat has gained new urgency in the era of COVID‐19, with viral‐induced endotheliitis increasing the incidence of thrombotic events [4]. This underscores a pressing need for therapeutic innovations that can effectively resolve thrombosis while minimizing harm. For acute thrombotic emergencies, the current standard of care relies on thrombolytic agents like urokinase (UK) and tissue plasminogen activator (rt‐PA) [5]. These drugs are potent, capable of dissolving the fibrin matrix of a clot to restore blood flow [6]. Yet, this potency comes at a high price. When administered systemically, these agents create a state of systemic fibrinolysis, leading to a substantial risk of severe bleeding, including intracranial hemorrhage. This danger is compounded by their short half‐life and lack of specificity, creating a narrow therapeutic window [7]. This fundamental paradox—the need for aggressive clot lysis versus the risk of catastrophic bleeding—highlights the critical need for a new paradigm: a targeted delivery strategy that can concentrate thrombolytic activity precisely where it's needed and nowhere else.

To address these challenges, research has shifted toward nanocarriers designed to shield thrombolytics and guide them to the clot [8, 9, 10, 11]. An especially promising platform is the ultrasound‐responsive microbubble, a clinically established contrast agent that can be engineered to carry a drug payload [12]. Applying localized ultrasound causes these microbubbles to rupture, enabling spatiotemporally controlled drug release, a concept validated in preclinical and clinical studies [13, 14]. However, two fundamental problems have hindered the translation of these “smart” delivery systems. First, they lack an active targeting mechanism to find the thrombus on their own. Second, like most synthetic nanomaterials, they are treated as foreign invaders and are quickly eliminated by the immune system, drastically reducing their therapeutic windows [15].

A powerful solution to both problems lies in biomimicry. By cloaking a nanocarrier with a natural cell membrane, the resulting “biomimetic” particle can evade immune clearance and inherit the unique surface functions of the source cell [16]. While membranes from platelets or red blood cells have been used to target the clot directly, this strategy can lead to undesirable off‐target effects on circulating cells [17, 18]. We hypothesized that a more specific approach would be to target not the clot itself, but the inflammatory microenvironment unique to it.

It is now understood that thrombosis and inflammation are deeply intertwined [19]. A thrombus releases a storm of chemokines, notably Monocyte Chemoattractant Protein‐1 (MCP‐1), which acts as a homing beacon for circulating monocytes. These monocytes express the C‐C chemokine receptor 2 (CCR2) and are actively recruited to the thrombus to mediate its resolution [20, 21]. This highly specific MCP‐1/CCR2 signaling axis, therefore, represents an ideal biological GPS for targeted drug delivery. By mimicking this natural homing process, a therapeutic agent could be delivered with exceptional precision, directly to the site of pathology.

**FIGURE 1:**
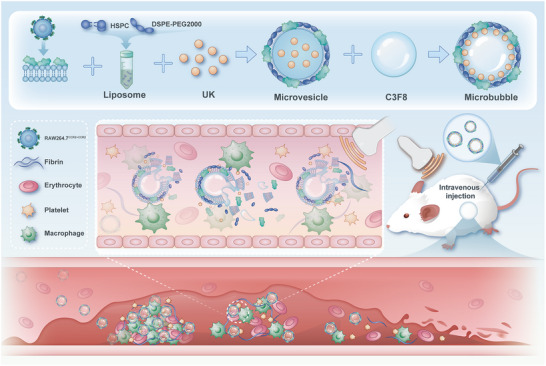
Design and mechanism of CCR2‐functionalized biomimetic microbubbles. Engineered macrophage membranes overexpressing CCR2 are used to construct urokinase‐loaded microbubbles (UK@CCR2/MBs). The microbubbles actively target the thrombus via the MCP‐1/CCR2 axis, where the localized ultrasound triggers payload release for precise thrombolysis.

In this study, we developed a biomimetic, ultrasound‐responsive microbubble platform designed for targeted thrombolysis across diverse vascular environments (Figure 1). We first engineered macrophage cells to overexpress CCR2 using lentiviral transfection. The membranes from these cells were then harvested and used to camouflage a liposomal core co‐encapsulating the thrombolytic agent UK and the phase‐change agent perfluoropropane. We hypothesized that upon intravenous injection, these biomimetic microbubbles would leverage the MCP‐1/CCR2 axis to actively home to the inflammatory microenvironment of a thrombus. Subsequent application of localized ultrasound would then trigger the disruption of the microbubbles, inducing immediate, on‐site release of UK to dissolve the clot. Through this strategy, we demonstrate a therapeutic approach that achieves both non‐invasive, targeted drug delivery and spatiotemporally controlled drug release, offering a promising new paradigm for the treatment of thrombosis.

## 2 Results

### 2.1 Characteristics of UK@CCR2/MB

**FIGURE 2:**
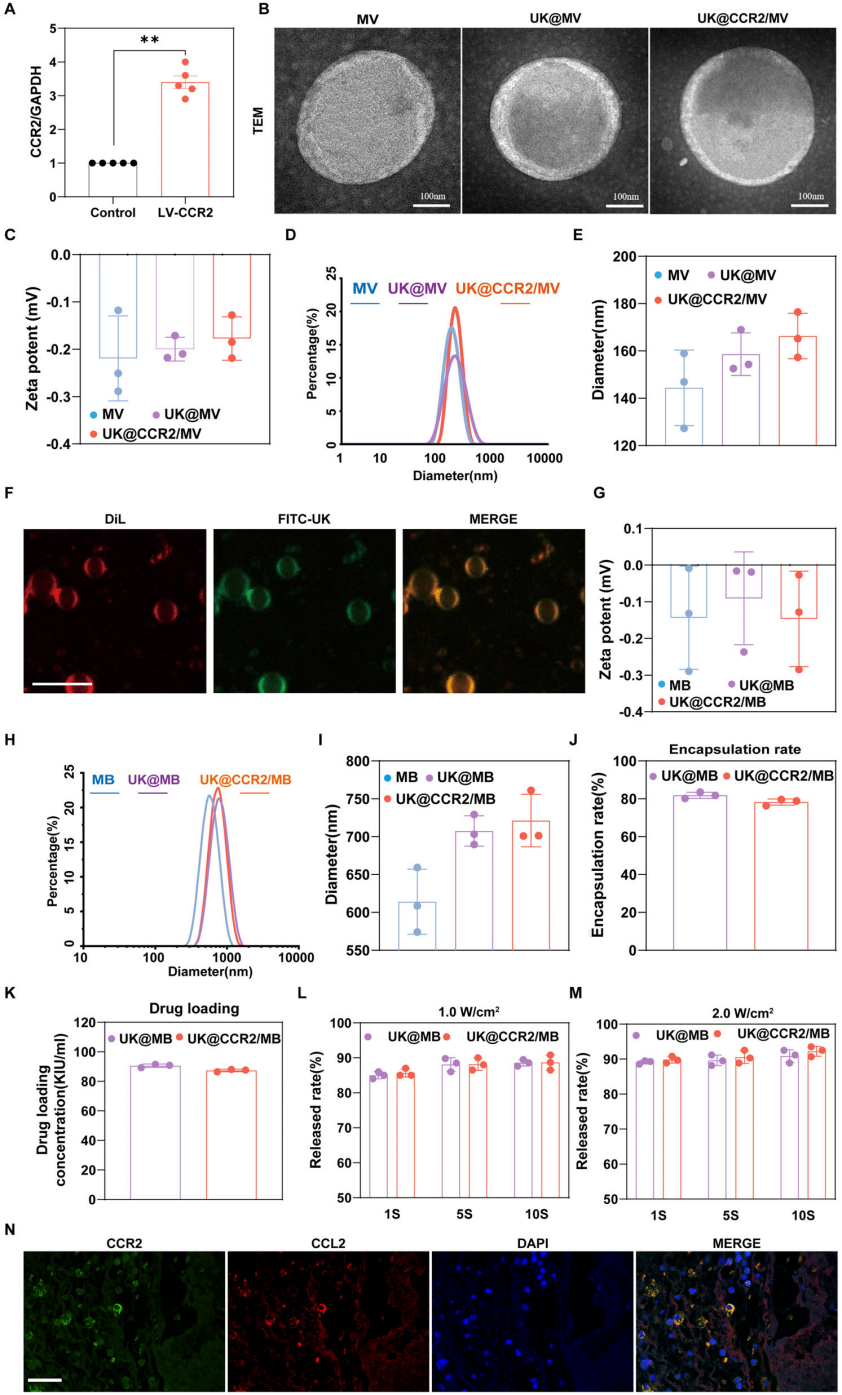
Characterization of urokinase‐loaded, ultrasound‐triggered microbubbles (UK@CCR2/MB) (A) Western blot analysis result demonstrated the overexpression of protein CCR2 in RAW246.7 cells (n = 5). (B) The morphology of MV, UK @ MV, and UK @ CCR2 / MV was observed by transmission electron microscopy, scale bar = 100 nm. (C–E) The charge and particle size data of MV, UK @ MV, and UK @ CCR2 / MV were analyzed (n = 3). (F) The microscopic images of UK @ CCR2 / MB were recorded using laser confocal microscopy, scale bar = 5 µm. (G–I) The charge state and particle size parameters of MB, UK @ MB, and UK @ CCR2 / MB (n = 3). (J, K) The encapsulation efficiency and drug loading concentration of UK @ MB and UK @ CCR2 / MB (n = 3). (L, M) The drug release rate of UK @ MB and UK @ CCR2 / MB under ultrasonic irradiation (frequency at 1.0 MHz, duty cycle at 50%, intensity at 1 W/cm^2^ or 2.0W/cm^2^) (n = 3). (N) Immunofluorescence results of CCR2 and CCL2 (also known as MCP‐1) expression in the thrombus, scale bar = 20µm. Data presented as mean ± SEM. Statistical significance was analyzed by unpaired two‐tailed Student's t‐test, *p*‐Values: ^**^ *p* < 0.01.

The inflammatory microenvironment‐targeting, macrophage membrane functionalized, urokinase‐loaded, ultrasound‐triggered microbubbles (UK@CCR2/MB) were successfully constructed (Figure 2; Figure S1). Immunostaining confirmed that macrophages expressed large amounts of MCP‐1 (ligand for CCR2) during thrombosis formation (Figure S1A). Considering the elevated level of MCP‐1 in the thrombosis and the natural receptor of MCP‐1, CCR2 was overexpressed on the membrane of macrophages by lentivirus transfection methods (Figure S1B,C). mRNA and protein levels of CCR2 were significantly increased, as confirmed by qPCR (Figure S1D) and western blot (Figures S1E; Figure 2A). Then, three types of cell membrane microvesicles (MV), namely MV, UK@MV, and UK@CCR2/MV, were primarily prepared. TEM scanning showed that the morphology of all MVs showed similar spherical structures (Figure 2B). The mean zeta potentials of MV, UK@MV, and UK@CCR2/MV were −0.22 mv, −0.2 mv, and −0.17mv, respectively (Figure 2C). Mean diameters of MV, UK@MV, and UK@CCR2/MV were 144.4 nm, 158.6 nm, and 166.3 nm, respectively (Figure 2D,E). Perfluoropropane(C_3_F_8_) was mixed into different MV suspensions under vacuum to form cell membrane microbubbles (MB). To detect successful microbubbles formation, Dil‐labelled cell membranes and FITC‐labelled UK were used. Immunofluorescence imaging showed that the shells of MBs were cell membranes (red) and UK(green), and the cores of MBs were C_3_F_8_ (Figure 2F). Compared to the corresponding MVs, MBs had slightly higher zeta potentials (−0.14, −0.09, −0.14mv for MB, UK@MB, and UK@CCR2/MB, respectively,) (Figure 2G) but nearly 5‐fold diameters (614, 707, and 721 nm, for MB, UK@MB, and UK@CCR2/MB, respectively) (Figure 2H,I), then detected by western blot (Figure S1F). The encapsulation rates of UK@MB and UK@CCR2/MB were approxmately 80% and 78%, respectively. The concentration of UK in UK@MB and UK@CCR2/MB was about 90.88 KIU/ml and 87.61 KIU/mL, respectively (Figure 2J,K). These microbubbles exhibited a spherical morphology, a nearly uniform size, and a well‐dispersed arrangement under the microscope, and they almost disappeared upon ultrasound stimulation (Figure S1G). Over 85% of the UK was rapidly released under 1, 5, and 10s stimulation with low‐frequency ultrasound (frequency at 1.0 MHz, duty cycle at 50%) with intensity at 1 W/cm^2^ or 2.0W/cm^2^ (Figures 2L,M). Together, these results suggest that UK@CCR2/MB with spherical structure, high drug encapsulation, and ultrasonic response is successfully fabricated and suitable for further investigations. To detect the targeted binding of UK @ CCR2/MB to MCP‐1 in the thrombus, fluorescence detection was performed. The results showed that CCR2 and MCP‐1 were coupled together (Figure 2N).

### 2.2 In vitro Thrombolytic Properties

**FIGURE 3:**
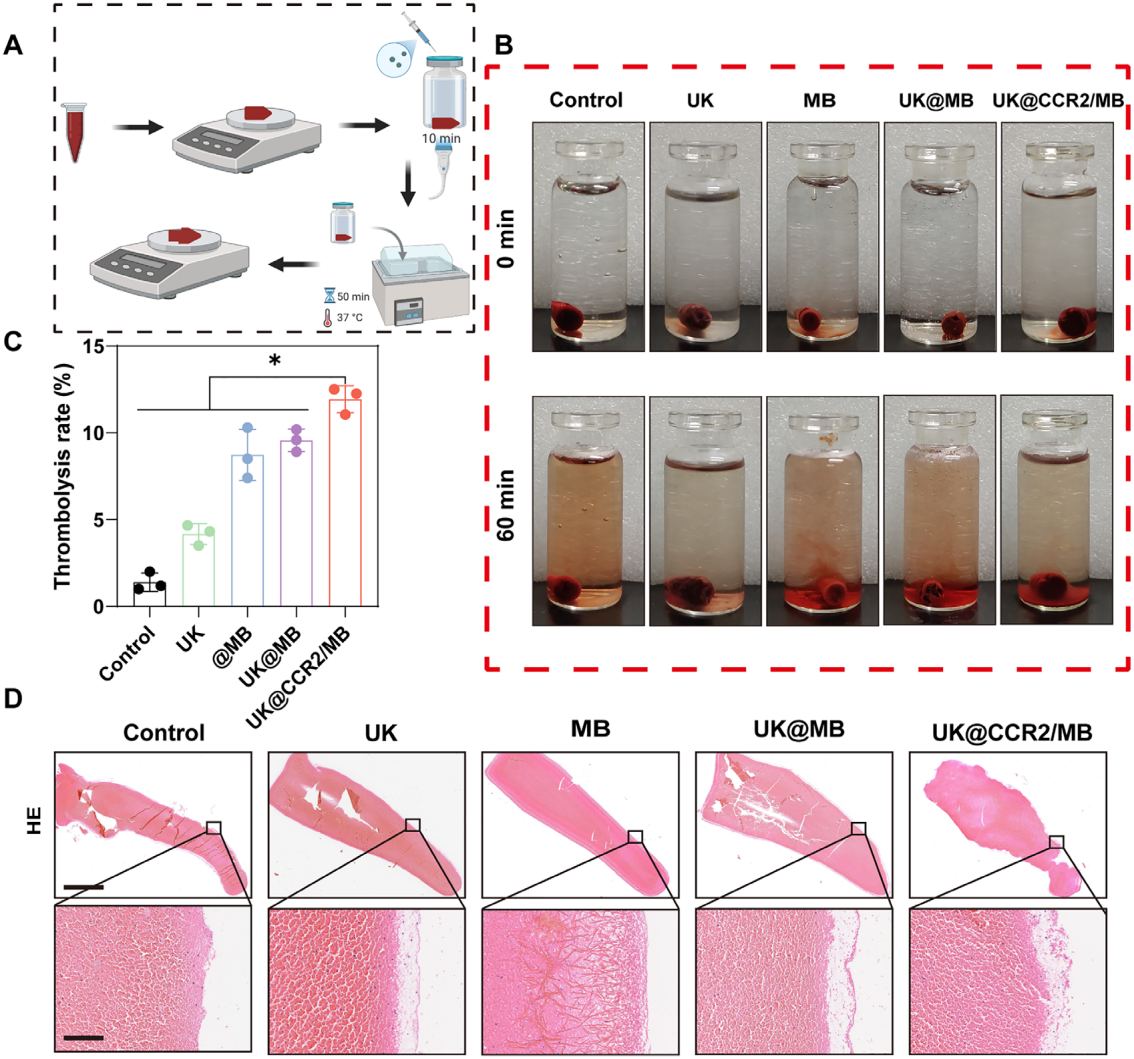
In vitro thrombolytic properties of UK@CCR2/MB (A) A schematic diagram of an in vitro thrombus treatment strategy. (B) The images of thrombus samples receiving different treatment conditions. (C) The thrombolysis rate of different treatments. (D) H&E staining of thrombus samples after different treatments, scale bar = 2.5mm and 100µm respectively. Data presented as mean ± SEM (n = 3). Statistical significance was analyzed by one‐way ANOVA testing followed by a Tukey post‐hoc test, *p*‐Values: * *p* < 0.05.

To evaluate the in vitro thrombolytic properties, a thrombotic thrombolysis model was established (Figure 3A). The different MBs were incubated with the same weight of thrombus in PBS, and the low‐frequency ultrasound (frequency at 1 MHz, duty cycle at 50%, intensity at 2 W/cm^2^) was applied for 10 min. After 50‐min incubation, visible partial thrombolytic components were deposited at the bottom, and the rest was weighed and histologically examined. Results showed that the red‐color deposition was the largest in the UK@CCR2/MB group (Figure 3B). Meanwhile, the thrombolysis rate was also the largest in the UK@CCR2/MB group (Figure 3C). H&E staining further confirmed that the surface of blood clots showed a pronounced, irregular, and loose surface in the UK@CCR2/MB group. Conversely, blood clots in the control and MB groups maintained relatively intact structures (Figure 3D). Thus, UK@CCR2/MB showed a strong thrombolytic ability under the stimulus of ultrasound in vitro.

### 2.3 In vivo Thrombolytic Efficacy for Deep Vein Thrombosis

**FIGURE 4:**
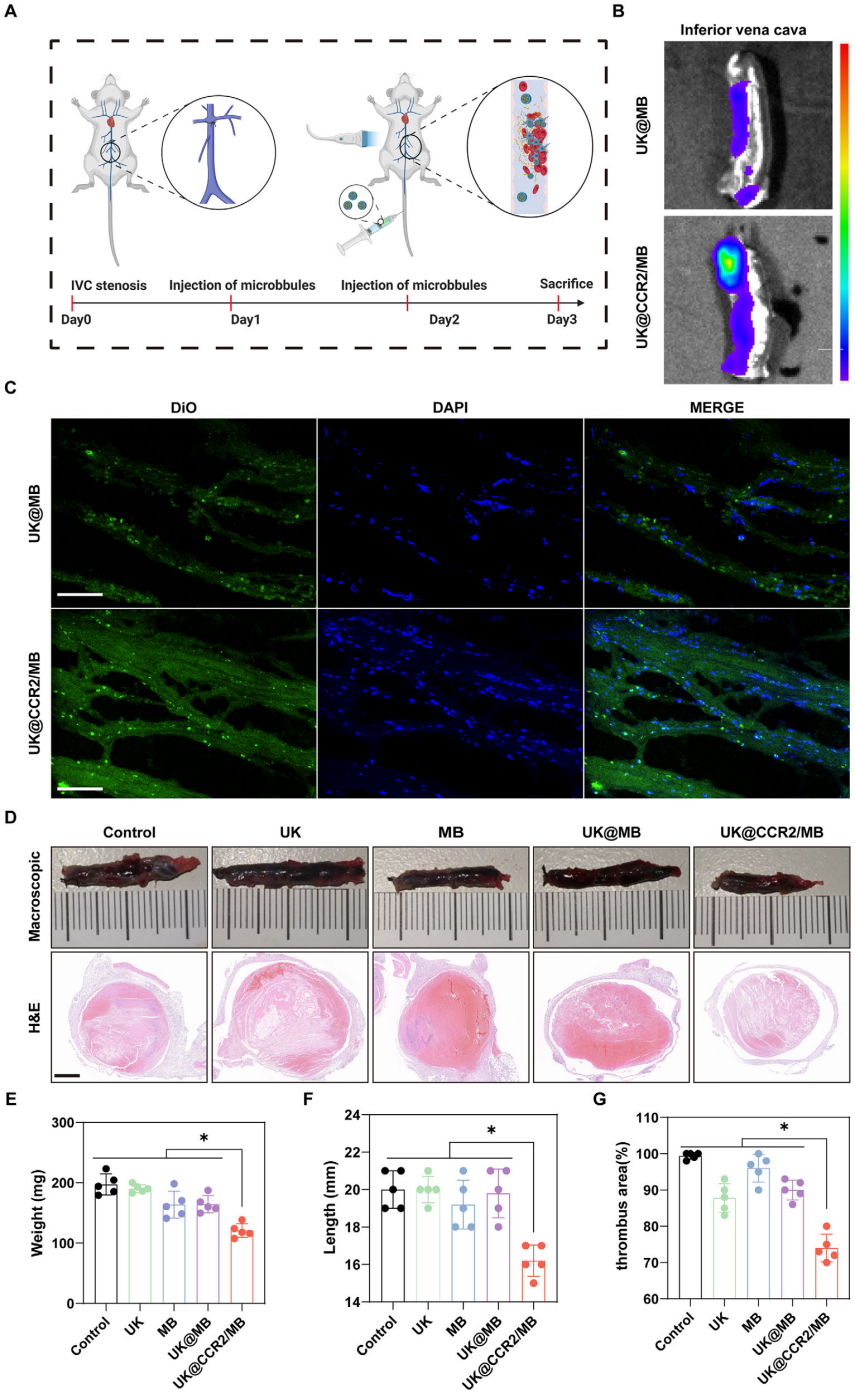
The thrombolytic efficacy of UK@CCR2/MB for deep vein thrombosis in vivo (A) Schematic diagram depicting the flow of inferior vena cava thrombosis treatment. (B) In vivo imaging of thrombus with different treatment methods. (C) Frozen section immunofluorescence of thrombus with different treatment methods, scale bar = 50 µm. (D)Macroscopic observation and H&E staining of inferior vena cava thrombosis after different treatments, scale bar = 500 µm. (E) Analysis of the weight of the thrombus after different treatments. (F) Analysis of the length of thrombus after different treatments was presented. (G) The proportion of thrombus area after different treatments. Data presented as mean ± SEM(n = 5). Statistical significance was analyzed by one‐way ANOVA testing followed by a Tukey post‐hoc test; *p*‐Values: *p < 0.05.

Venous thrombosis, including deep vein thrombosis (DVT) and pulmonary embolism (PE), occurs in approximately 1 person per 1000 each year [22, 23, 24]. As a standard animal model of deep vein thrombosis, the inferior vena cava ligation thrombosis mouse model was established to evaluate the thrombolytic efficacy of different MBs (Figure 4A). After intravenous injection of the same amounts of MBs, the site of blood clots in the UK@CCR2/MB group displayed a stronger fluorescence intensity than that of the UK@MB group (Figure 4B). Then, frozen section fluorescence showed more fluorescent MBs in the UK@CCR2/MB group (Figure 4C), demonstrating the enhanced ability to target thrombosis. Among all the groups, the UK@CCR2/MB group had the lowest length and weight of thrombosis (Figure 4D). The weight of thrombosis in the UK@CCR2/MB group was about 110 mg, which is significantly lower than that of the other groups (Figure 4E). The length of thrombosis was decreased by 20% in the UK@CCR2/MB group, compared to other groups (Figure 4F). Moreover, the H&E staining and quantitative analysis showed that the percentage of thrombus area in the UK@CCR2/MB group was 74.5%, and significantly lower than that of any other group (Figure 4D,G). These results confirmed the targeting ability and strong efficacy of UK@CCR2/MB for the treatment of deep vein thrombosis.

### 2.4 In vivo Thrombolytic Efficacy in the Artery Thrombosis Model

**FIGURE 5:**
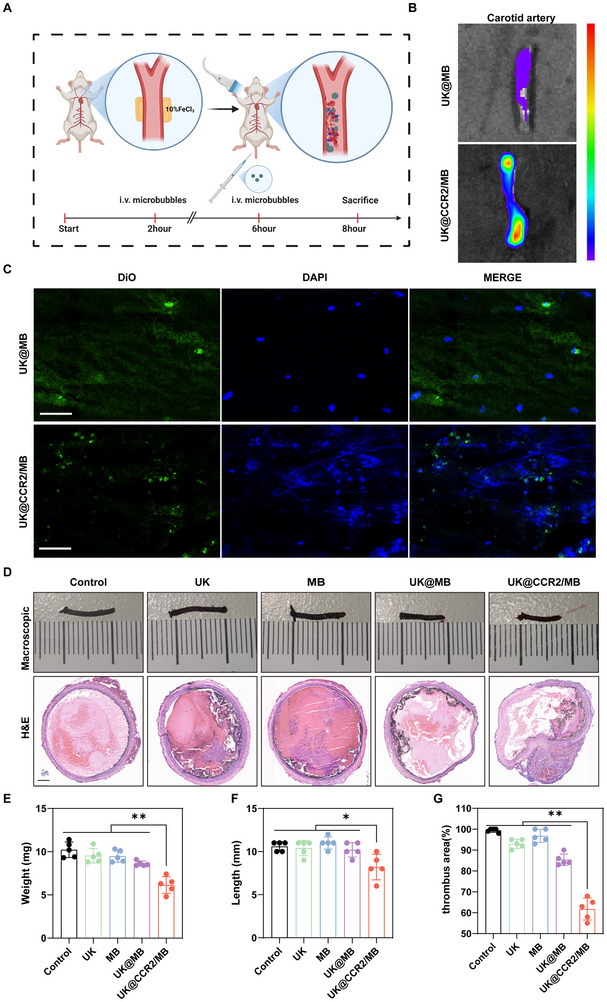
The thrombolytic efficacy of UK@CCR2/MB for carotid artery thrombosis in vivo (A) The flow chart of carotid artery thrombosis treatment. (B) Targeting effects of different treatment methods on carotid artery thrombosis. (C) The frozen section fluorescence of different treatments, scale bar = 50µm. (D) Macroscopic observation and H&E staining of carotid artery thrombosis after different treatments, scale bar = 100µm. (E) Analysis of the weight of carotid artery thrombosis after different treatments. (F) Analysis of the length of thrombus after different treatments of carotid thrombosis. (G) Analysis of the proportion of thrombus area after different treatments of carotid thrombosis. Data presented as mean ± SEM(n = 5). Statistical significance was analyzed by one‐way ANOVA testing followed by a Tukey post‐hoc test; *p*‐Values:*p < 0.05; **p < 0.01.

Artery thrombosis could occur in the coronary, cerebral, carotid, or lower extremity arteries and is known as ischemic heart disease, ischemic stroke, and peripheral arterial occlusive disease, placing a heavy burden globally [25]. The classic FeCl_3_‐triggered rat carotid arterial thrombosis model was established to investigate the therapeutic benefits of MBs in arterial thrombolysis (Figure 5A). In the carotid artery thrombosis model, the UK@CCR2/MB group had stronger fluorescence intensity in the thrombosis sites than the UK@MB group (Figure 5B). Then, frozen section fluorescence detection was performed. Compared with the UK @ MB group, the UK @ CCR2 / MB group showed more fluorescent MBs, demonstrating the enhanced ability to target arterial thrombosis(Figure 5C). The weight and length of thrombosis were also the lowest in the UK@CCR2/MB group (Figure 5D–F). Moreover, the histologic analysis showed that the percentage of thrombus area in the UK@CCR2/MB group was significantly lower than in other groups (Figure 5D,G). These results confirmed the targeting ability and strong efficacy of UK@CCR2/MB for the treatment of arterial thrombosis in vivo.

### 2.5 Thrombolytic Efficacy for Microvascular Thrombi in Vivo

**FIGURE 6:**
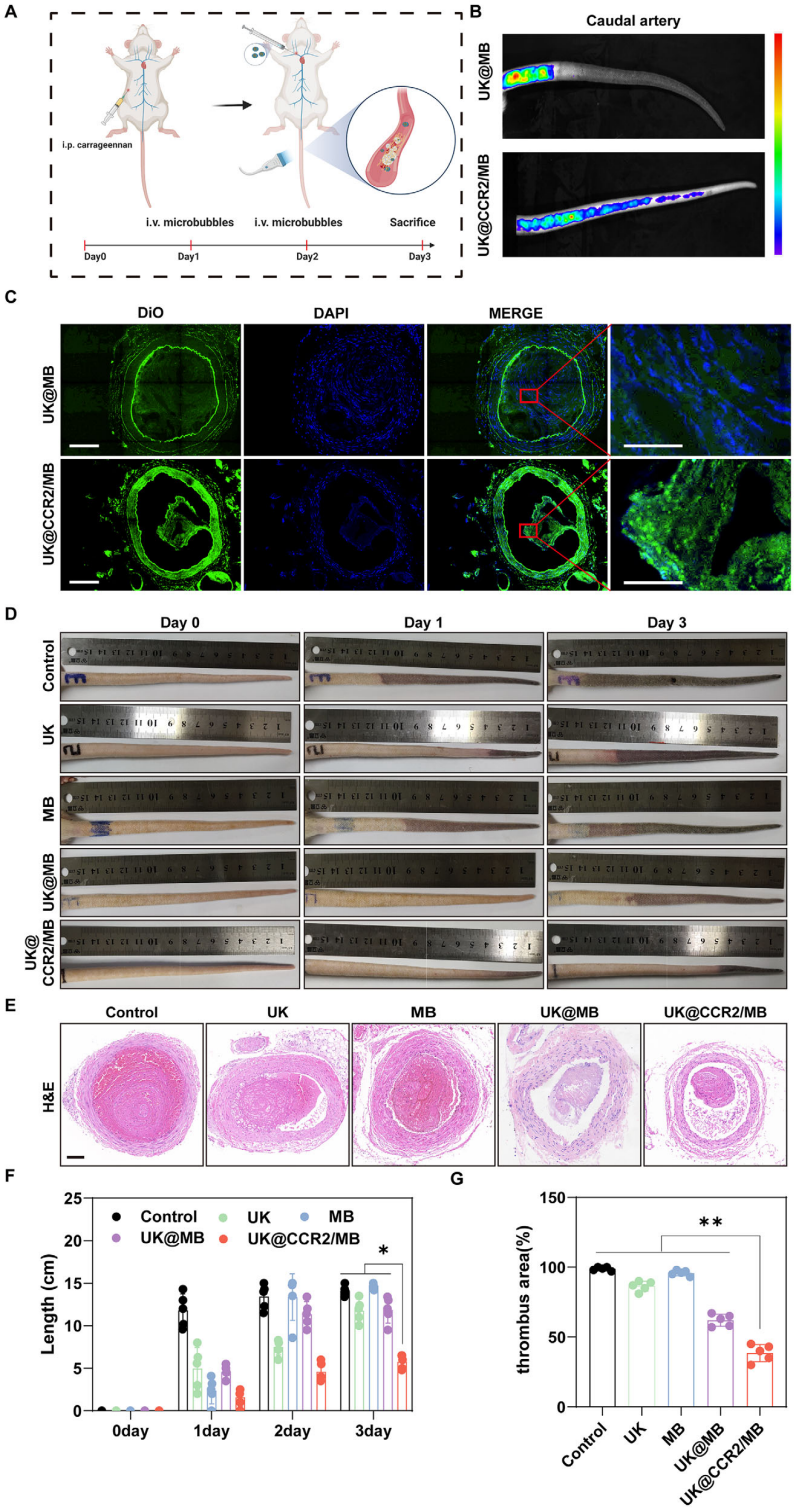
The thrombolytic efficacy of UK@CCR2/MB for microvascular thrombi in vivo (A) The flow chart of the caudal artery thrombosis animal model and treatment. (B) Targeting effects of different treatment methods on caudal thrombosis. (C) The fluorescence detection results of frozen section were presented, scale bar = 20 µm,50 µm, respectively. (D) Macroscopic observation of caudal artery thrombosis after different treatments. (E) H&E staining of caudal artery thrombosis after different treatments, scale bar = 100 µm. (F) Analysis of the length of caudal artery thrombosis after different treatments. (G) Analysis of the proportion of thrombus area after different treatments of caudal thrombosis. Data presented as mean ± SEM (n = 5). Statistical significance was analyzed by one‐way ANOVA testing followed by a Tukey post‐hoc test; *p*‐Values:*p < 0.05; **p < 0.01.

Microvascular thrombi were the common pathological cause of circulatory dysfunction and organ failure in COVID‐19 patients and critically ill patients [26, 27]. In this study, a rat caudal artery thrombosis model was established to simulate microvascular thrombi (Figure 6A). Following targeted treatment, the fluorescence intensity was stronger and longer in the rat tails of the UK@CCR2/MB group than the UK@MB group, suggesting the UK@CCR2/MB had improved targeting and thrombolytic ability (Figure 6B). Then, frozen section fluorescence detection was performed, which showed more fluorescent MBs in the UK@CCR2/MB group (Figure 6C). After treatment, the length of the thrombus was the shortest in the UK@CCR2/MB group, followed by the UK@MB group (Figure 6D). The average thrombus length of the UK@CCR2/MB group was approximately half that of the other groups (5.4 cm vs 11.0‐15.0cm, p < 0.05, Figure 6F). H&E staining demonstrated that the UK@CCR2/MB group exhibited the largest recanalization proportions, but blood vessels of other groups were almost occupied by a thrombus (Figure 6E). The stereological analysis showed that the percentage of thrombosis area in the UK@CCR2/MB group was about 25%, which was the lowest among all the groups (Figure 6G). Therefore, UK@CCR2/MB had marked thrombolytic efficacy for microvascular thrombi in vivo.

### 2.6 Biocompatibility and Safety

**FIGURE 7:**
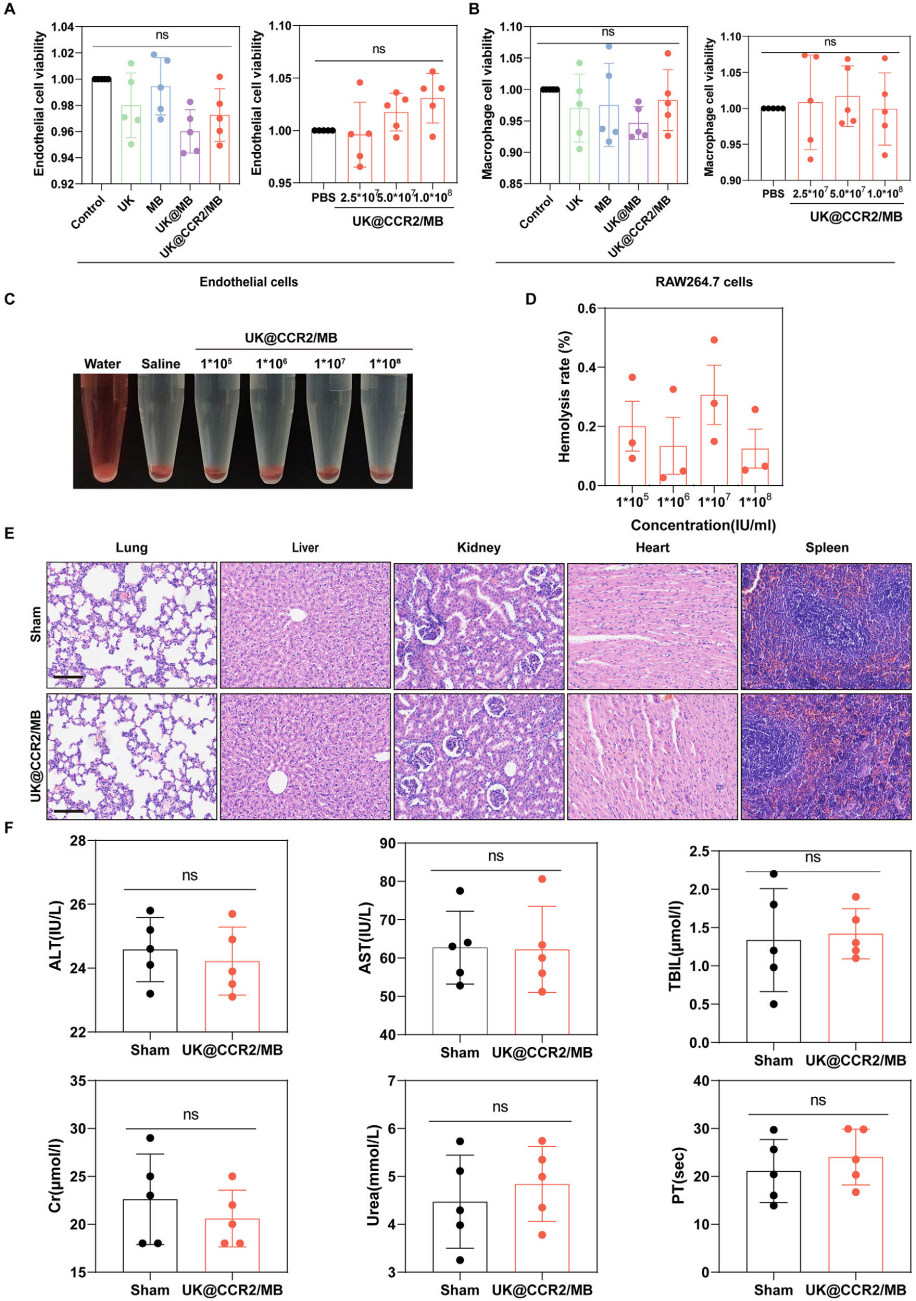
Biocompatibility and safety of the UK@CCR2/MB in vitro and in the rat model of deep vein thrombosis. (A,B) The CCK‐8 results of endothelial cells and RAW264.7 cells (n = 5). Statistical significance was analyzed by one‐way ANOVA testing followed by a Tukey post‐hoc test. (C,D) The hemolysis effect of different concentrations of UK@CCR2/MB (n = 3). Statistical significance was analyzed by one‐way ANOVA testing followed by a Tukey post‐hoc test. (E) HE staining of lung, liver, kidney, heart, spleen of the inferior vena cava thrombosis animals after UK@CCR2/MB treatment, scale bar = 100 µm. (F) The serum level of ALT, AST, TBIL, Cr, Urea, and PT of the inferior vena cava thrombosis animals after UK@CCR2/MB treatment (n = 5). Statistical significance was analyzed by unpaired two‐tailed Student's t‐test. Data presented as mean ± SEM. ns, no significant.

In order to evaluate the toxicity of MBs, endothelial cells and RAW264.7 cells were treated with UK, @ MB, UK @ MB, and different concentrations of UK @ CCR2 / MB. The results of the CCK‐8 assay showed that the MBs caused no obvious damage to cells, and different concentrations of UK@CCR2/MB didn't reduce the viability of endothelial cells and macrophages, indicating remarkably low in vitro cytotoxicity (Figure 7A,B). To evaluate the hemolytic properties in vitro, various concentrations of UK@CCR2/MB were incubated with rat‐derived erythrocytes, while deionized water and 0.9% saline were used as controls. Under ultrasonics, all the concentrations of UK@CCR2/MB did not exhibit any apparent hemolysis (Figure 7C), and the observed hemolysis rates were far below 0.5% (Figure 7D). In the rat model of deep vein thrombosis, there were no obvious structural alterations in the hearts, lungs, livers, kidneys, and spleens of rats administered with UK@CCR2/MB when compared to the sham group (Figure 7E). Additionally, the liver, kidney, and coagulation functions in the UK@CCR2/MB group were comparable to those in the sham group (Figure 7F). Similarly, the structures and functions of internal organs remained unchanged in the animal model of the artery (Figure S2A,B) and microvascular (Figure S2C,D) thrombosis. None of the animals had bleeding complications. Taken together, the UK@CCR2/MB has excellent biocompatibility and safety in vitro and in vivo.

## 3 Discussion

Current thrombolytic therapies are plagued by severe bleeding risks and a lack of specificity [28]. Our study presents a novel solution: an inflammation‐targeting, ultrasound‐activated microbubble (UK@CCR2/MB). We confirmed that these biomimetic microbubbles use the MCP‐1/CCR2 axis to home to venous, arterial, and microvascular thrombi, where localized ultrasound triggers on‐demand urokinase release. This strategy offers a non‐invasive, highly targeted, and effective approach to thrombolysis, representing a significant improvement in safety and precision.

The design of UK@CCR2/MB was rationalized to overcome the limitations of prior technologies. We integrated microbubbles with ultrasound—a non‐invasive clinical tool—to amplify the therapeutic effect beyond what sonothrombolysis alone can achieve [29, 30]. To solve the critical issues of immune clearance and non‐specific delivery that hinder simple microbubbles, we employed a biomimetic cell membrane coating. We specifically chose macrophage membranes over other cell types, like platelets or erythrocytes, because they can be genetically engineered [31, 32, 33]. This allowed us to overexpress the CCR2 receptor, hijacking a natural inflammation‐homing pathway to grant our platform an active and highly specific targeting capability. This combination of biomimetic camouflage, genetic targeting, and triggered release creates a powerful and sophisticated therapeutic system, as demonstrated by its efficacy across diverse thrombosis models.

Our study distinguishes itself by targeting the inflammatory microenvironment of the thrombus rather than its structural components. Conventional approaches targeting platelet or fibrin markers risk off‐target binding [34], whereas the MCP‐1/CCR2 axis offers a uniquely specific target. MCP‐1 is highly expressed locally at the thrombus but not systemically, creating a precise homing beacon for natural clot resolution [19, 35]. By engineering macrophage‐membrane microbubbles to express CCR2, we harnessed this biological pathway for targeted drug delivery, a strategy validated by both molecular analysis and in vivo imaging.

The clinical viability of this platform depends on its safety. We confirmed that our optimized formulation prevented microbubble aggregation. Furthermore, we conducted a thorough safety evaluation. UK@CCR2/MB proved to be highly biocompatible, showing no hemolysis or cytotoxicity in vitro. Most importantly, even when tested across three distinct thrombosis models in vivo, the platform induced no bleeding complications, organ toxicity, or adverse effects on hematological and biochemical parameters. This combination of a novel, inflammation‐specific targeting mechanism and a demonstrated high safety profile establishes our platform as a promising candidate for future clinical translation.

## 4 Conclusion

This study reported a novel treatment strategy for venous, arterial, and microvascular thrombosis based on the inflammatory microenvironment‐targeting, macrophage membrane functionalized, urokinase‐loaded, ultrasound‐triggered microbubbles (UK@CCR2/MB). CCR2 was strongly expressed on the macrophage membrane and subsequently coated on the liposomes to target the elevated MCP‐1 in thrombosis. The thrombolytic drug urokinase and ultrasound‐responsive perfluoropropane were encapsulated in the biomimetic microbubble. Under the local ultrasound stimulation, the size of perfluoropropane rapidly expanded, and microbubbles exploded to release urokinase and resolve thrombosis. UK@CCR2/MB displayed strong thrombolytic ability and high safety in vitro and in various thrombosis animal models. Therefore, these ultrasonic macrophage membrane microbubbles not only non‐invasively deliver the thrombolytics to the thrombosis sites but also precisely control the drug release to provide a novel therapeutic approach for thrombosis.

## 5 Methods

### 5.1 Materials

Urokinase (Southern Medicinal Company, Nanjing, China), hydrogenated soy phosphatidylcholine (HSPC) (Avanti Polar Lipids, Alabaster, AL, USA), di‐stearoyl phosphatidylcholine‐polyethylene glycol 2000 (DSPE‐PEG2000) (Ruixi Biotechnology, Xi'an, China), perfluoropropane (Wan Shui Special Gases Co. Ltd, Shanghai, China), cholesterol (Aoweituo Medical Technology Co. Ltd, China), and Fluorescein Isothiocyanate (FITC), and Dil Fluorescent Dye (Nanjing Star Ye Biotechnology Co. Ltd, China) were used. Furthermore, fetal bovine serum and DMEM medium (Gibco, USA), penicillin and streptomycin (Dingguo Biological), and lentivirus CCR2 products (Shanghai Jikai Company) were used in this study. Total RNA extraction kit, reverse transcription kit, and qPCR detection kit were obtained from Novazan. Rabbit anti‐CCR2 (ab273050) (Abcam, USA) and rabbit anti‐GAPDH antibody (14C10) were purchased from Cell Signaling Technology (Danvers, MA, USA). The BCA Protein Quantitation Kit was obtained from the Senbei Jia Company in China.

### 5.2 Animals

The healthy male Sprague Dawley (SD) rats, aged about 8 weeks and weighing between 180 and 220 g, were provided by the Animal Experiment Center of the Army Medical University of the People's Liberation Army of China and raised in the SPF level breeding environment of the center. All experimental procedures were carried out in accordance with the approval requirements of the Animal Ethics Committee (AMUWEC20237074).

### 5.3 Cell Culture and Lentiviral Transfection

RAW 264.7 cell lines (ATCC RRID: CVCL‐0493) were cultured in DMEM (Dulbecco's Modified Eagle Medium) medium containing 10% fetal bovine serum (FBS) and 1% penicillin and streptomycin at 37°C and 5% CO_2_. In the experiment, RAW 264.7 cells were first evenly distributed in a six‐well culture plate (1 × 10^5^ cells per well), and then on the second day, based on the target virus replication index (MOI), lentivirus containing overexpression of the CCR2 gene was introduced for gene transfection. After a 48‐h transfection process, the cell culture medium was removed and replaced, and puromycin was added to screen the transfection effect. After selecting stable and well‐growing cells, they were allowed to proliferate. Cells were then stored frozen until they were used.

### 5.4 Cell Membrane Extraction

After cell collection, the operating procedure of the cell membrane extraction kit was followed. Briefly, an appropriate amount of cell membrane extraction agent A was added and kept for 15 min on an ice bath. After rupturing the cells using the ice thawing method, the clear liquid was separated and collected by slow centrifugation (at 4°C, with a force of 700×g for 10 min). Next, high‐speed centrifugal separation was achieved (at 4°C, centrifuged at 14000×g force for 30 min), and after discarding the clear liquid, the cell membrane sediment was obtained.

### 5.5 Preparation of Membrane Microvesicles and Microbubbles

HSPC and DSPE‐PEG2000 were prepared in appropriate proportions (the molar ratio is 9:1) to make an empty liposome suspension through a polycarbonate filter membrane (400 nm). Diluted the cell membrane with 5 mM Dil (used for cell membrane labeling) in DMSO at a ratio of 1:1000 and added it to the cell membrane solution using a polycarbonate filter membrane (400 nm). 0.5 mL of empty liposome suspension and 1ml of high‐concentration urokinase solution (100000 units/ml) were mixed, and then emulsification was performed using ultrasound in an ice bath (300 W, 10 min). Corresponding cell membrane solution was added at a ratio of 1:50, and emulsification was performed using ultrasound (power 100 W, 10 min). The subsequently obtained finer and more uniform microvesicles were obtained through filtration using polycarbonate film (200 nm). After filtration and sterilization, 0.9 mL of cell membrane vesicle solutions from various groups were tightly packed and sealed in a sterile container. A vacuum pump was used to remove the air, followed by adding perfluoropropane (C_3_F_8_) gas to completely replace the air. The mixture was stirred with a silver mercury stirrer for 40 s to prepare microbubbles.

### 5.6 Characterization of Microvesicles and Microbubbles

The shape and size of MVs were examined through transmission electron microscopy (TEM). The morphological characteristics and size of MBs were evaluated by optical microscopy. The zeta potential and mean size of MVs and MBs were determined by using a dynamic laser scattering particle sizer. High‐resolution images of Dil‐labeled MBs were obtained using a laser confocal scanning microscope to determine their shape and actual size.

### 5.7 Drug Loading Rate Detection of Microbubbles

FITC‐labeled urokinase was dialyzed in a dialysis bag to remove free FITC. After preparation of urokinase solutions of different concentrations, a standard curve was plotted with the activity unit of urokinase as the x‐axis and the UV absorption OD value of FITC as the y‐axis. A certain amount of microbubbles were taken into a 2ml EP tube and diluted with PBS to 500ul. Coupling agent was applied on the outside of the EP tube cover, and the EP tube was then inverted to the ultrasound probe. Ultrasound irradiation (frequency at 1.0 MHz, duty cycle at 50%, intensity at 2.0W/cm^2^) for 1 min was used for the complete bursting of microbubbles. The UV absorption value of the solution after ultrasound irradiation was measured at a wavelength of 498 nm. The FITC‐UK content (W_UK1_) in the microbubbles were calculated using a standard curve. The mass of FITC‐UK initially added during microbubble preparation is represented by W_UK0_, and the formula for calculating the encapsulation efficiency of UK is as follows:

$$\mathrm{Encapsulation}\;\mathrm{rate}\;\left(\%\right)=W_{\mathrm{UK}1}/W_{\mathrm{UK}0}\times 100\%$$

### 5.8 In vitro Release of Microbubbles

A total of 10ul cell membrane‐loaded UK microbubbles was taken to a 2ml EP tube and diluted with PBS to 1ml. The outer side of the EP tube was covered with coupling agent, and the EP tube was inverted to the ultrasound probe. Under different ultrasound conditions, the above microbubbles were centrifuged at 3000 r/min, for 3 min. And the lower solution was taken to measure the UV absorption value at a wavelength of 498 nm. The FITC‐UK content C_T_ in the lower solution was calculated using standard curves.

$$\mathrm{UK}\;\mathrm{release}\;\mathrm{rate}\;\left(\%\right)=\left(C_{T}/C_{0}\right)\;\times \;100\%$$

Among them, C_T_ represents the total amount of drug cumulative release at a certain time point, and C_0_ represents the total amount of UK encapsulated in microbubbles.

### 5.9 Thrombolysis Experiment in vitro

1mL of blood taken from Sprague Dawley (SD) rats was poured into a 1.5mL EP tube and left to stand for 4 h to form a thrombus at 37°C. Next, the formed thrombus was placed in a glass vial containing 5mL PBS. 100 µL of MB, UK @ MB, and UK @ CCR2 / MB were added, and then irradiated for 10 min with ultrasound (frequency at 1.0 MHz, duty cycle at 50%, intensity at 2.0W/cm^2^). Subsequently, the vials were placed in a water temperature of 37°C and further incubated for 50 min. The thrombolysis effect was evaluated by the weight change of the thrombus. H&E staining was performed on the processed thrombus samples.

### 5.10 Cytotoxicity Assay

RAW 264.7 and HUVEC were seeded in 96‐well microplates and incubated for 24 h at 37°C. Subsequently, 100ul of different MBs (UK, MB UK@MB) as well as various concentrations of UK@CCR2 (2.5 × 10^7^, 5 × 10^7^, 1 × 10^8^) were cultured for 12 h on this basis. After washing with PBS, 100 microliters of serum‐free medium containing 10% CCK‐8 reagent was added to each culture well. Cells were further incubated at 37°C for 2 h, and then the OD value of the cells was measured at a wavelength of 450 nm.

### 5.11 Inferior Vena Cava Thrombosis Experiment

Before the operation, the SD rats fasted for 12 h and were anesthetized via intraperitoneal injection of 1% pentobarbital sodium. They were groomed by shaving their abdomens and placed in a supine position on the operating table, where the abdominal area was disinfected. The abdominal cavity was then progressively opened, and the intestinal contents were squeezed to the left side of the operator and placed on gauze moistened with saline solution. The inferior vena cava was exposed and isolated (below the renal artery), then all branches were ligated. The isolated inferior vena cava was ligated with a 7‐gauge syringe needle and 4‐0 silk medical suture, followed by the prompt removal of the needle and closure of the abdominal cavity [36]. The rats were randomly assigned into five groups: Control, UK, MB, UK@MB, and UK@CCR2/MB (n = 5). The rats were injected with PBS, UK, MB, UK@MB, and UK@CCR2/MB (each 100 µL) by tail vein at 24 and 48 h postoperative, and then followed by 10 min of ultrasound irradiation (frequency at 1.0 MHz, duty cycle at 50%, intensity at 2.0W/cm^2^). After 72 h, blood samples and inferior vena cava thrombi were collected from the SD rats for serological assays, live imaging, and H&E staining.

### 5.12 Carotid artery Thrombosis Experiment

Under the condition of fasting for 12 h, anesthesia was administered by intraperitoneal injection of 1% pentobarbital sodium, followed by shaving of neck hair. The SD rats were then fixed supine on the operating table and disinfected. The right carotid artery was gradually dissected. The arterial area was wrapped with a 10mm × 10mm filter paper soaked in 10% FeCl_3_ for 10 min. After thoroughly rinsing with physiological saline, the incision was closed [37]. The rats were randomly divided into Control, UK, MB, UK‐MB, and UK‐CCR2/MB groups (n = 5). At 2 h and 6 h after surgery, the rats were injected with PBS, UK, MB, UK‐MB, and UK‐CCR2/MB (100 µL) through the tail vein and subjected to 10 min of ultrasonic irradiation (frequency at 1.0 MHz, duty cycle at 50%, intensity at 2.0W/cm^2^). Blood samples and carotid thrombus samples were collected at 8 h after surgery for serological analysis, in vivo imaging, and H&E staining.

### 5.13 Caudal Artery Thrombosis Experiment

After a 12‐h fasting period, each rat received an intraperitoneal injection of carrageenan at a dosage of 20 mg/kg [38]. The SD rats were randomly divided into Control, UK, MB, UK‐MB, and UK‐CCR2‐MB groups (n = 5). At 24 and 48 h after injection of carrageenan, the rats received PBS, UK, MB, UK‐MB, and UK‐CCR2‐MB (100µl) correspondingly via jugular vein injection, followed by ultrasonic irradiation (frequency at 1.0 MHz, duty cycle at 50%, intensity at 2.0W/cm^2^) for 10 min. After 72 h, the blood serum and carotid artery thrombi were harvested for serologic assays and histopathological H&E staining.

### 5.14 Live Imaging Detection

After inhaling isoflurane anesthesia, samples are placed on a stage and parameters (excitation wavelength 470 nm, emission wavelength 520 nm, exposure time 0.6s) were set for real‐time imaging. The imaging software was used to analyze imaging data.

### 5.15 H&E Staining and Serum Analysis

The samples were fixed with 4% paraformaldehyde for 72 h, then dehydrated in a gradient ethanol solution, embedded in paraffin for fixation, and finally dewaxed with xylene I and II until they reached a transparent state. After completing the H&E staining process, the tissue was examined for pathological changes under an optical microscope, and corresponding image collection was performed. Serum samples were sent to the Testing Center of Southwest Hospital for ALT, AST, TBIL, Cr, Urea, and PT analysis.

### 5.16 Hemolysis Test

Fresh red blood cells were obtained from the blood samples of SD rats and then washed with physiological saline for several times. An equal amount of 100ul of red blood cells was diluted with different concentrations of UK@CCR2 /MB solution (ranging from 1 × 10^5^ to 1 × 10^8^, prepared in physiological saline). The pure physiological saline group served as the negative control, and ultrapure water as the positive control. After being incubated in a 37°C water bath for 3 h, the supernatant of each experimental group was collected. Then, the absorbance was recorded using a measuring instrument with a wavelength of 540 nm. The following formula was used to calculate the hemolysis rate. Hemolysis rate (%) = ((Sample Absorption ‐ Negative Control Absorption)/(Positive Control Absorption ‐ Negative Control Absorption)) × 100.

### 5.17 Statistics

All statistical evaluations were performed using GraphPad Prism 8.0 software (San Diego, CA, USA). Continuous variables are expressed as mean ± SEM. For normally distributed data sets with equal variances, unpaired two‐tailed student's t‐test was used to determine significant differences between the two groups, and one‐way ANOVA testing followed by a Tukey post‐hoc test was carried out across three or more groups. In all cases, significance was defined as *p* ≤ 0.05.

## Author Contributions

Buying Li and Changjin Lu contributed equally to this work, designing and conducting the experiments, collecting data, and preparing the manuscript. Shijie Gao and Dandan Chen assisted with data analysis. Xue Heng and Kibret Mequanint provided critical experimental design support. Haisheng Li, Gaoxing Luo, and Malcolm Xing supervised the project, secured funding, and contributed to conceptual development, manuscript editing, and revisions. All authors reviewed and approved the final manuscript.

## Conflicts of Interest

The authors declare no conflict of interest.

## Supporting information




**Supporting File**: advs74372‐sup‐0001‐SuppMat.docx.

## Data Availability

The data that support the findings of this study are available from the corresponding author upon reasonable request.
