# Supplementary material for: Targeted Thrombolysis via CCR2‐Engineered Macrophage‐Mimicking Microbubbles Safely Ablates Venous, Arterial, and Microvascular Thrombosis
Source: Adv Sci (Weinh). 2026 Mar 13;13(23):e24002. doi: 10.1002/advs.202524002 (PMC13104076; doi:10.1002/advs.202524002)
Supplement: Supplementary file 1 — Supporting File: advs74372‐sup‐0001‐SuppMat.docx. [file ADVS-13-e24002-s001.docx]

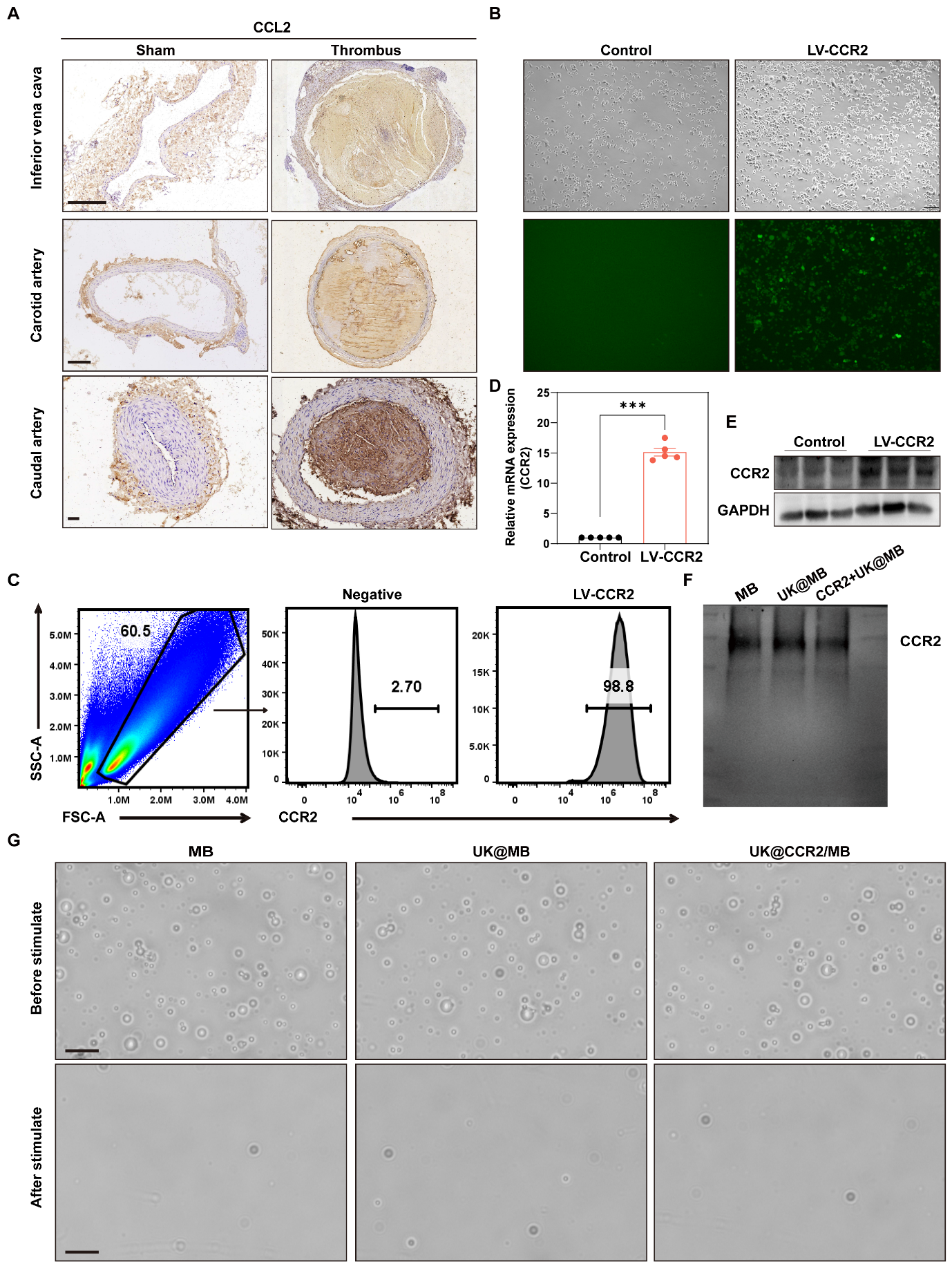


**Figure S1 Characterization of urokinase-loaded, ultrasound-triggered microbubbles (UK@CCR2/MB)**

A) Immunohistochemical staining of CCL2 in the thrombus ( inferior vena cava, carotid artery, and caudal artery, scale bar= 200μm; B) Light microscope and fluorescence images of RAW264.7 cells transfected by CCR2-expressing lentivirus, scale bar= 200μm. C) Flow cytometry examination of LV-CCR2 positivity rate in RAW264.7 cells. D) Quantitative PCR detection of CCR2 mRNA expression in RAW264.7 cells. E) Western blot analysis of CCR2 protein expression in RAW264.7 cells. F) Western blot analysis of CCR2 protein expression in MB, UK @ MB, and UK @ CCR2/MB; G)The microscopic images of MB, UK @ MB, and UK @ CCR2/MB, scale bar=10μm. Data presented as mean ± SEM (n=5). Statistical significance was analyzed by unpaired two-tailed Student’s t-test; *p*-Values:*** *p*<0.001.

**
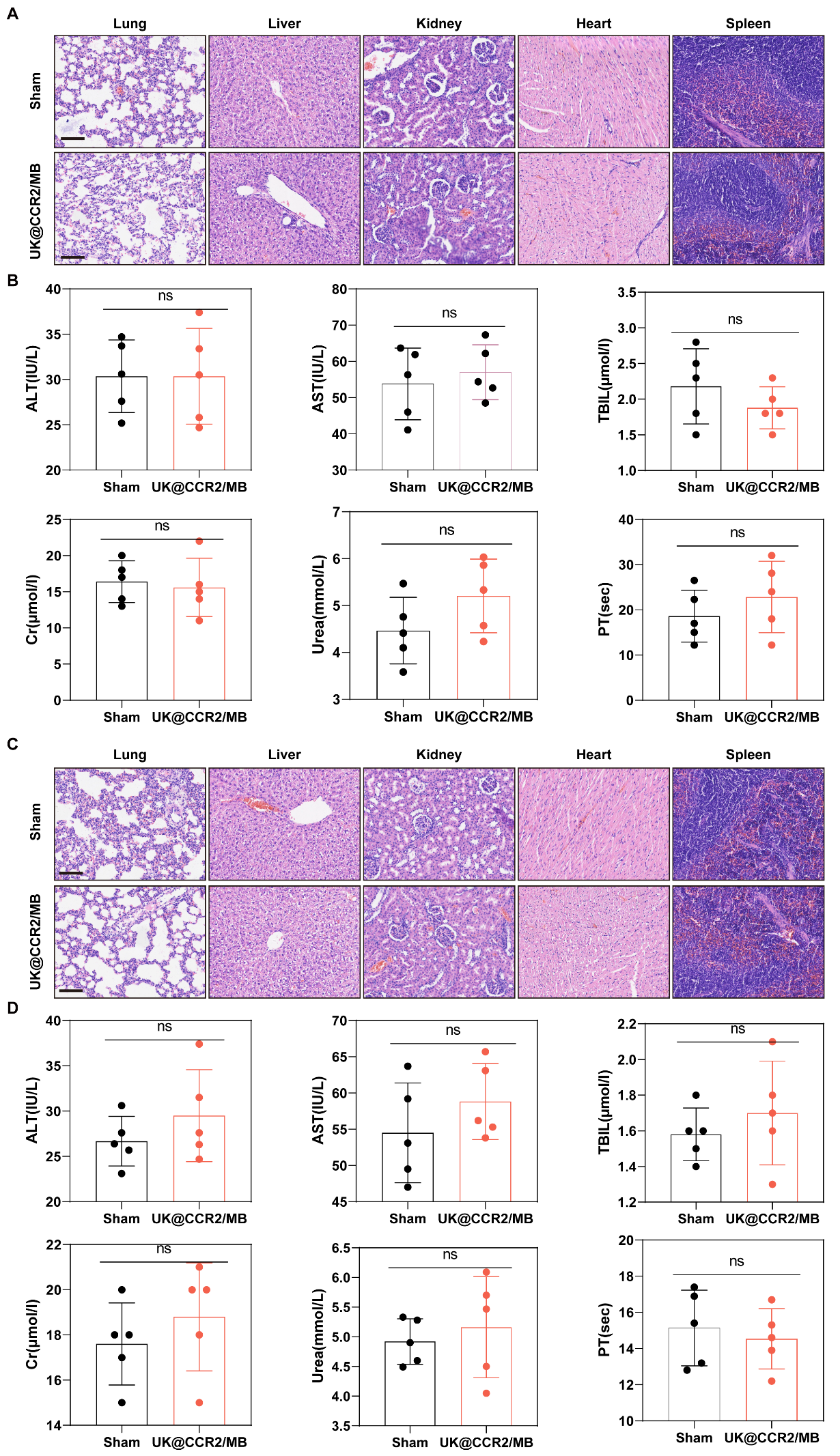
**

**Figure S2. Safety of the UK@CCR2/MB in the rat model of carotid artery thrombolysis and microvascular thrombi**

A) H&E staining of the heart, liver, spleen, lung, and kidney of the carotid artery thrombosis animals after UK@CCR2/MB treatment, scale bar = 100μm. B) The serum levels of ALT, AST, TBIL, Cr, Urea, and PT of the carotid artery thrombosis animals after UK@CCR2/MB treatment. C) H&E staining of the heart, liver, spleen, lung, and kidney of the caudal artery thrombosis animals after UK@CCR2/MB treatment, scale bar = 100μm. D) The serum level of ALT, AST, TBIL, Cr, Urea, and PT of the caudal artery thrombosis animals after UK@CCR2/MB treatment. Data presented as mean ± SEM(n=5). Statistical significance was analyzed by unpaired two-tailed Student’s t-test; ns, no significant.
